# Natural Bioactive Compounds of *Sechium* spp. for Therapeutic and Nutraceutical Supplements

**DOI:** 10.3389/fpls.2021.772389

**Published:** 2021-12-21

**Authors:** María Isabel Iñiguez-Luna, Jorge Cadena-Iñiguez, Ramón Marcos Soto-Hernández, Francisco Javier Morales-Flores, Moisés Cortes-Cruz, Kazuo N. Watanabe

**Affiliations:** ^1^Postgrado de Innovación en Manejo de Recursos Naturales, Colegio de Postgraduados, San Luis Potosí, Mexico; ^2^Interdisciplinary Research Group at Sechium edule in Mexico, A.C., Texcoco, Estado de México, Mexico; ^3^Programa de Botánica, Colegio de Postgraduados, Montecillo, Mexico; ^4^Centro Nacional de Recursos Genéticos-INIFAP, Tepatitlán, Mexico; ^5^Tsukuba Plant Innovation Research Center, University of Tsukuba, Tsukuba, Japan

**Keywords:** cucurbitacins, flavonoids, fruit, cancer, diabetes, endemic species

## Abstract

Natural products are in great demand because certain secondary metabolites (SMs) are sources of antioxidants, flavorings, active substances, or anticancer agents with less aggressiveness and selectivity, among which triterpenes and flavonoids are of importance because they inhibit carcinogenesis. For *Sechium* spp. P. Br. (chayotes), there is scientific evidence of antiproliferative activity that has occurred when cancer cell lines have been treated with this fruit. In order to compare future therapeutic designs and identify new and ancestral characteristics, triterpenes and flavonoids were determined in contrasting *Sechium* genotypes. The obtained data were analyzed *via* a cladistics approach, with the aim of identifying the characteristics and state of phytochemicals and genetic variables. The concentrations of flavonoids and triterpenes were determined, and a more complex composition of secondary metabolites was found in the wild types as compared to their domesticated genotypes. Bitter fruits contained a higher number of SMs, followed by those with a neutral and sweet flavor. A cladogram showed the differentiation of the three groups based on the flavor of the fruits. The diversity of SMs decreases in evolutionary terms, in response to domestication and environmental adaptation. Therefore, genotypes can be feasibly selected based on fruit flavor for gross-breeding, and cytotoxicity can be reduced without losing possible therapeutic effects.

## Introduction

The use of medicinal plants is based on empirical knowledge that has been preserved for generations, with the aim of improved or recovered health (Palma-Tenango et al., 2017). In Mexico, it is estimated that there are 5,000 species of plants with healing properties (Villaseñor, 2016); similarly, different active substances have been isolated from these, sometimes obtaining more than one per plant (Molina-Mendoza et al., 2012). These substances correspond to secondary metabolites (SMs), which are biomolecules that enable plants to survive, adapt, and reproduce when they are threatened by predators or stress conditions (Piasecka et al., 2015).

Recently, SMs have become increasingly important in the food, industrial, cosmetic, textile, and pharmaceutical sectors (Bourgaud et al., 2001), and approximately 200,000 SMs have been identified (Croteau et al., 2000). Some species of the genus *Sechium* P. Browne (Cucurbitaceae) are found among plants with broad diversity and complex composition as reported in Mexico, whose fruits are known as chayotes (Lira et al., 1999; Cadena-Iñiguez et al., 2011). The SMs confer particular characteristics to this species, thereby creating morpho-biochemical differences that affect the phenotype, and there is evidence in *Sechium* of morpho-biochemical changes in edible types. Among the phyto compounds that have aroused particular interest are triterpenes, due to their antiproliferative properties. These have been evaluated with different *in vitro* and *in vivo* models, in an attempt to define their mechanism of action and effects when they are used as cancer cell treatments (Vega et al., 2006; Petronelli et al., 2009; Bishayeen et al., 2011; Aguiñiga-Sánchez et al., 2017). Flavonoids are metabolites identified in *Sechium*, and there are reports of their antineoplastic activities (Deschner et al., 1993; Manach et al., 1996; Salazar-Aguilar et al., 2017).

In the development of anticancer compounds from natural sources, various active substances have been extracted and commercialized, such as vincristine, camptothecin, and taxol. However, some of these have shown toxic effects or non-selective activities, resulting in the elimination of every cell with a high proliferation rate, such as lymphocytes and hair cells (Vega et al., 2006; Jacobo-Herrera et al., 2016). Under this premise, genetic improvement has begun with certain types of chayote because antineoplastic activity has been demonstrated through bioassays in the WEHI-3, HeLa, P388, J774, and L929 cell lines, among other principal ones (Cadena-Iñiguez et al., 2013; Aguiñiga-Sánchez et al., 2015, 2017; Salazar-Aguilar et al., 2017). In bone marrow mononuclear cells, DNA fragmentation of malignant cells was found, revealing the selective activity of chayote extracts. The advantages cited, in addition to being a genus that integrates cultivable genotypes having production yields exceeding 64 t ha^–1^ per year (SIAP, 2019), confer importance to chayote as a source of biomass for active substances with pharmacological objectives.

Agrobiodiversity includes wild relatives of domesticated genotypes or in the process of domestication (Convenio de Diversidad Biológica [CDB], 2016), however, there is a risk of genetic loss or erosion due to changes in consumer preference, displacement by new cultivars and habitat fragmentation. The intra- and interspecific complexes of agrobiodiversity contain biological variants with great potential for the extraction of SMs for diverse uses and are economically important for the pharmaceutical, health and food industries (Roskov et al., 2015). It is estimated that the diversity of vascular plants in Mexico ranges from 22,000 to 31,000 species, of which about 4,000 have medicinal use (Leon-Lobos et al., 2012).

Based on the differences in the phytochemical profiles of *Sechium sp*., an attempt has been made to help explain the causes of the morphobiochemical changes observed in the morphotypes. However, there is interest in developing genotypes with medium-term yields in the field (biomass) to recover SMs of therapeutic interest that increases the pharmacological options and dietary supplements. Starting from the hypothesis that domesticated genotypes have lost many SMs, while wild types can be highly toxic, an evaluation of contrasting and underutilized genotypes of *Sechium sp*. was carried out, in order to identify SMs for pharmacological and nutraceutical use, in order that it contributes to the design of new varieties by genetic crossing to obtain bioactive natural products, and to favor their revaluation and importance as components of agrobiodiversity that reduce the risk of loss.

## Materials and Methods

### Materials

**FIGURE 1:**
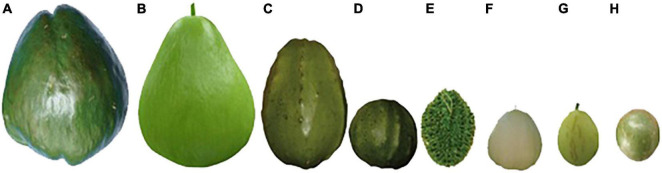
Morphotypes of *Sechium* spp. evaluated. **(A)** *S. edule* var. *nigrum xalapensis*, **(B)** *S. edule* var. *virens levis*, **(C)** *S. compositum* (accession 11), **(D)** S. *compositum* (accession 751), **(E)** *S. edule* wild-type, **(F)** *S. edule* var. *albus levis*, **(G)** *S. edule* var. *albus minor*, **(H)** *S. edule* var. *nigrum minor*. The reference metric scale is 1 cm per square.

*Sechium* spp. fruits of genotypes with contrasting flavors were chosen that were considered representative of the contrasts (Cadena-Iñiguez et al., 2011; Table 1; Figure 1). The fruits evaluated were harvested at a stage of horticultural maturity (Table 1), and all of them came from the national gene bank of *Sechium edule* in Mexico (BANGE*Se*) (19° 08′ 48″ N, 97° 57′ 00″ W). All accessions registered their entry into the germplasm bank in 2005 and have been maintained under agroclimatic conditions and equal management.

**TABLE 1:** Main characteristics of the accessions of *Sechium* spp. based on fruit color and flavor (*n* = 27).

Genotype	Accession	Color	Flavor	Status
*S. edule var. albus levis*	769	yellow	sweet	Semi-domesticated
*S. edule var. albus levis*	761	yellow	sweet	Semi-domesticated
*S. edule var. albus minor*	330	yellow	sweet	Semi-domesticated
*S. edule var. nigrum minor*	681	dark green	neutral	Semi-domesticated
*S. edule var. virens levis*	273	light green	neutral	Domesticated
*S. edule var. nigrum xalapensis*	530-a	dark green	neutral	Domesticated
*Sechium edule*	653	dark green	bitter	Wild type
*Sechium compositum*	11	dark green	bitter	Wild type
*Sechium compositum*	751	dark green	bitter	Wild type

### Extraction Process

The fruits were washed, cut, weighed, and then placed in a drying oven (BLUE-M, Electronic Company/Blue Island, IL, United States) with an air flow at 45°C. After drying for 72 h, all parts of the fruits (exocarp, mesocarp, spines, and seed) were ground in a mill (Hamilton Beach, United States). A continuous extraction of the dry ground material was carried out with methanol (Cadena-Iñiguez et al., 2013; Aguiñiga-Sánchez et al., 2017). From the ground material, 2.5 g were weighed for each accession and submerged in 80% methanol at a 1:10 ratio in 50 mL Falcon tubes. The samples were homogenized, placed in an ultrasonic bath (Branson B-220 50/60 Hz) at room temperature, and were then subjected to two cycles of 10 min sonication and 5 min of rest between each cycle. Subsequently, the tubes were centrifuged at 3000 *g* for 5 min. and the supernatant was collected in 2 mL amber vials, and stored in the fridge before analysis by HPLC (Aguiñiga-Sánchez et al., 2017).

### Determination of Cucurbitacins and Flavonoids

Based on a previously described methodology (Salazar-Aguilar et al., 2017), cucurbitacins and flavonoids were analyzed by high-performance liquid chromatography (HPLC), where 20 mg of extract was weighed per sample, dissolved with 1 mL of HPLC-grade methanol (Sigma-Aldrich, St. Louis, MO, United States), and then filtered using a nylon membrane acrodisc with a diameter of 0.45 μm (Merck, Millipore, Germany).

Cucurbitacins were analyzed through a symmetry shield C18 column (4.6 × 250 mm) (Waters, Spain) *via* an isocratic analysis. The mobile phase used was in isocratic mode with water, methanol, and acetonitrile (50:30:20 v/v), and the injection flow rate was 1 mL min^–1^ at a pressure of 179 bars, with all the samples being at a temperature of 25°C. The total volume injected per accession evaluated was 20 μL. Finally, the identification of cucurbitacins was performed using two wavelengths (λ1 235 nm and λ2 254 nm). As a reference standard, cucurbitacins D, I, B, and E were used (Sigma-Aldrich, United States).

In the case of flavonoids, a hypersil ODS column (125 × 40 mm) was used (Hewlett-Packard, United States). with a gradient of (A) H_2_O adjusted at pH 2.5 with trifluoroacetic acid, and (B) acetonitrile 0–10 min, in the following mixtures A:B 85:15 for 20 min, and A:B 65:35 for 25 min. The following parameters were used: flow at 1 mL/min at 30°C, detection wavelengths, 254, 316, and 365 nm; injection volume, 20 μL and analysis time, 25 min. The standards used were rutin, florizidin, myricetin, quercetin, naringenin, florentin, and galangin (Sigma-Aldrich, United States).

### DNA Extraction for Amplified Fragment Length Polymorphism Bands

In order to demonstrate an approach to the relationship between secondary metabolites and genetic expression, DNA was extracted from the young leaves of previously evaluated accessions (Shagai-maroof et al., 1984) and characterized by AFLPs (Vos et al., 1995). A double digestion was applied to 500 ng of DNA with *Eco*RI/*Mse*I restriction enzymes (10 and 2.5 U/μg, respectively) at 37°C for 4 h. After evaluating the restriction fragments by electrophoresis in 0.8% agarose for 70 min at 80 V, the ligation began by adding 10 μL of a mixture of adaptors at 5 mg kg^–1^ to the T4 DNA ligase enzyme at a final concentration of 1 U.

For the pre-amplification, 4 μL of the ligation product was combined with 21 μL of a mixture containing starters *Mse*I + C (5 μM), *Eco*RI + A (5 μM), and 10.5 μL of Sigma REDTaq ^®^ ReadyMix™ PCR Reaction Mix and was finally adjusted to a volume of 25 μL with Type I water. The thermocycler was programmed at 94°C for 30 s, 56°C for 60 s, and 72°C, for 25 cycles. The result of this PCR was corroborated in 0.8% agarose gel for 60 min at 80 V, and then, the pre-amplification reaction was diluted with 80 μL of Type I water.

For the selective amplification, 3.0 μL of the aforementioned dilution were taken, adding 3 μM *Eco*RI + ACC marked with FAM and 5 μM *Mse*I + CAT, and adjusting to a final volume of 10 μL with 1× SIGMA REDTaq ^®^ ReadyMix™ PCR Reaction Mix. The thermocycler was programmed at 95°C for 5 min, followed by 35 cycles (95°C for 40 s, 54°C for 40 s, and 72°C for 90 s) and final elongation at 72°C for 40 min.

The product of the amplification was observed in a 6% polyacrylamide gel that underwent electrophoresis at 200 V for 3 h. The gels were dyed with silver nitrate as soon as the fragments were visualized, and then, a dilution (1:10) of the PCR-selective and formamide products (Hi-Di™ Formamide, United States) was prepared and evaluated in a capillary sequencer 3500 XL Genetic Analyzer (ThermoFisher Scientific, United States) along with the fragment analysis running model (FragmentAnalysis50_POP7xl_2, Fragment_Analysis_PA_Protocol). Using the amplification data (size of the fragment), a binary matrix of presence and absence (1/0) was generated through the Gene Mapper 4.1 software (GeneMapper, Applied Biosystems).

### Statistical Analysis

The data were analyzed *via* a cladistics approach with WinClada 1.00.08 (2002) (WinClada v.1.00.08, 2020). The applied analysis was heuristic, with the Bootstrap/Jackknife tests, which generated the index of consistency and stability, respectively (Felsenstein, 1985; Lanyon, 1985).

## Results

### Phytochemical Screening

The relationship between fresh and dry weight and extract yield showed that on average, the genotypes evaluated were composed of 73.85% water. As previously noted, fruits with a yellow epidermis (*albus minor*, *albus levis*) showed a lower percentage (52.27%), and those with the highest water content were the green fruits with a neutral flavor (82.40%), followed by the wild types accounting for 79.76%. In the case of the extract yield, 52.98% was found for genotypes of neutral flavor, while the *albus* (yellow) yielded 39.26%, and finally the wild fruits of bitter flavor accounted for 37.27% (Table 2).

**TABLE 2:** Relationship between fresh, dry weight, and percentage yield of extracts from fruits at horticultural maturity stage of *Sechium* spp. (*n* = 27).

Fruit flavor/accession									
**Variable**	**Sweet**			**Neutral**			**Bitter**		
	**769**	**761**	**330**	**681**	**273**	**530-a**	**11**	**751**	**653**
Fresh weight (g)	84.94	34.3	10.44	9.63	360	390	555	147.24	119.41
Dry weight (g)	27.6	19.1	4.7	3.3	37.9	29.7	80.6	27.3	33.00
Water (%)	67.5	55.68	54.98	65.33	89.5	92.38	85.47	81.45	72.36
Dry matter (%)	32.49	44.31	45.02	34.26	10.5	7.61	14.52	18.54	27.63
Extract (g)	1.15	1.08	0.71	1.07	1.29	1.61	0.94	1.01	0.85
Total yield (%)	46.04	43.29	28.47	42.94	51.6	64.37	37.44	40.52	33.91

High-performance liquid chromatography analysis revealed the presence of cucurbitacins I, D, B, and E, and in terms of flavonoids, rutin, myricetin, and floretin were found in all the genotypes, while galangin was present in all except for *virens levis* and *nigrum xalapensis* (neutral flavor). Quercetin was found in six of the nine genotypes, and was absent in *albus levis*, *virens levis*, and *nigrum xalapensis*. Naringenin was observed in *albus minor* and the two wild ones of *S. compositum*. Finally, florizidin was identified in *virens levis* and in accession 11 of *S. compositum* (Tables 3, 4).

**TABLE 3:** Yield of cucurbitacins obtained from fruits at horticultural maturity stage of contrasting flavor of *Sechium* spp. (*n* = 27).

Genotype	mg g^–1^			
	CuD	CuI	CuB	CuE
*S. edule var. albus levis*	1.17	4.27	1.27	0
*S. edule var. albus levis*	4.77	3.52	0.46	1.73
*S. edule var. albus minor*	12.71	1.93	1.52	0
*S. edule var. nigrum minor*	1.06	5.84	0.27	0
*S. edule var. virens levis*	0.95	4.04	0.62	0
*S. edule var. nigrum xalapensis*	13.44	5.60	0.73	0
*Sechium compositum*	3.00	4.70	1.96	0
*Sechium compositum*	8.35	17.47	4.37	0
*Sechium edule* wild type	1.04	3.10	0.18	0

*CuD = cucurbitacin D; CuI = cucurbitacin I; CuB = cucurbitacin B; CuE = cucurbitacin E.*

**TABLE 4:** Flavonoid content obtained from fruits at horticultural maturity stage of contrasting flavor of *Sechium* spp. (*n* = 27).

Genotype	mg g^–1^							
	Ru	Fz	My	Qu	Na	Ft	Ap	Ga
*S. edule var. albus levis*	0.26	0.00	0.78	0.00	0.00	0.09	0	0.46
*S. edule var. albus levis*	0.13	0.00	0.55	0.00	0.00	0.10	0	0.43
*S. edule var. albus minor*	0.25	0.00	0.69	0.01	0.16	0.05	0	0.48
*S. edule var. nigrum minor*	0.13	0.00	0.27	0.01	0.00	0.06	0	0.44
*S. edule var. virens levis*	0.23	0.08	1.09	0.00	0.00	0.11	0	0.00
*S. edule var. nigrum xalapensis*	0.08	0.00	0.77	0.00	0.00	0.15	0	0.00
*Sechium compositum*	0.48	0.11	0.19	0.03	0.56	1.42	0	5.80
*Sechium compositum*	1.20	0.00	1.46	0.21	1.59	2.19	0	12.32
*Sechium edule* wild type	0.82	0.00	1.10	0.09	0.00	0.06	0	0.51

*Ru = rutin; Fz = florizidin; My = myricetin; Qu = quercetin; Na = naringenin; Ft = floretin; Ap = apigenin; Ga = galangin.*

The genotypes with higher concentrations of cucurbitacin D (CuD) and CuE were yellow fruits (*albus minor, albus levis*), while higher amounts of CuB and CuI were found in bitter chayotes (*S. compositum* and *S. edule*). In the case of CuD, the highest concentration was found in *nigrum xalapensis*, followed by *albus minor* and *S. compositum*. In terms of CuI, the highest value was identified in *S. compositum* and *nigrum minor*, and in the case of CuB, the highest concentration was for *S. compositum* and *albus minor*. The only material that contained CuE was *albus levis* (accession 761).

High-performance liquid chromatography analysis for flavonoids showed that there were seven to eight standards in the evaluated genotypes, and no apigenin was reported. Rutin, myricetin, quercetin, and galangin were identified from the groups of flavonols, florizidin and floretin were identified from the chemical group of dihydrochalcones, and only naringenin came from flavones. In general, the highest concentration of flavonoids was quantified in bitter genotypes, followed by chayotes of neutral flavor. Only rutin and naringenin flavonoids were found in higher quantities in yellow fruits (*albus levis* and *albus minor*) (Table 4).

### Combination of Phytochemical Variables and Polymorphic Bands of Amplified Fragment Length Polymorphisms

Figure 1 shows the parsimonious arrangement of the combination of phytochemical variables and polymorphic bands of AFLPs (one thousand repetitions), signaling a reconstruction of inheritable similarities, where bitter chayotes of dark green color with spines were the genotypes most proximal to the root of the monophyletic tree, corresponding to ancestral characteristics. The cladogram shows the three groups distinguished by the flavor of the fruits. The first was formed by bitter genotypes, placing *S. edule* as the root taxon, followed by the two accessions of *S. compositum*. Similarly, the second group was formed by green chayotes of neutral flavor, and the last group was formed by those of yellow fruit of sweet flavor. Accordingly, and under a cladistics view, an evolutionary arrangement was marked, suggesting that the bitter flavor is an ancestral characteristic that changes (decreases) due to differences in secondary metabolites and associated polymorphic bands. The first case was observed in the group of bitter chayotes, where an absence of the 16 (77 pb) band was found in the ancestral traits (dark circles) of *S. edule* and *S. compositum* (accession 11), as well as the mean concentration of CuD in accession 751 of *S. compositum*.

It is difficult to consider abrupt changes in the genotypes of a species in the same agroclimatic region of distribution. However, small differences attributed to the same environment can possibly be identified. For example, even when accession 11 and 751 are *S. compositum* and come from the same region (Chiapas, Mexico), the first one comes from the evergreen tall forest, and the second comes from ruderal conditions without vegetation, which can timely mark modifications in the phenotype. Plasticity is a trait that allows plants to carry out morphological and biochemical adjustments, and especially for this case, accession 11 is of pyriform fruit, while accession 751 is round and small (Figure 1). The second group included genotypes with neutral-flavor fruits and corresponded to those with the highest consumption and commercial manipulation, indicating the presence of cucurbitacin B, rutin, and myricetin, which are classified as apomorphic traits (new). Finally, the genotypes *albus minor*, *albus levis*, and even *nigrum minor*, considered as semi-domesticated, of low consumption, and conserved in family backyards, exhibited six traits (Table 5), half of which corresponded to phytochemicals with mean values in terms of concentration of cucurbitacins D and E, such as the symplesiomorphic trait (ancestral and shared with the other genotypes), and the presence of band 54 (426 pb), as an autapomorphic trait (new).

**TABLE 5:** Plesiomorphic and apomorphic traits, from the analysis of 12 biochemical variables and 48 polymorphic bands of AFLPs, from *Sechium* spp. (Phytochemical trait 0–11; AFLP bands: 12–59).

Genotype/accession	Trait/status of trait per clade	Internal clade
*Sechium edule* wild type 653	0/4, 2/5, 4/5, 7/3, 8/3, 11/4, 47/1, 53/1	4/1, 7/0, 11/0,
*Sechium compositum 11*	0/2, 2/4, 5/2, 8/2, 12/0, 16/0	
*Sechium compositum 751*	0/5, 2/3, 6/3, 28/1, 30/1, 31/1, 41/1, 48/1, 59/1	
*S. edule var. nigrum xalapensis 530a*	1/2, 2/2, 4/5, 5/1	
*S. edule var. virens levis 273*	0/2, 11/1, 54/0	27/0, 33/0,43/0
*S. edule var. nigrum minor 681*		0/2, 11/1, 54/0
*S. edule var. albus minor 330*		
*S. edule var. albus levis 761*	0/3, 3/1, 29/0	13/0
*S. edule var. albus levis 769*	11/2, 54/1	

**FIGURE 2:**
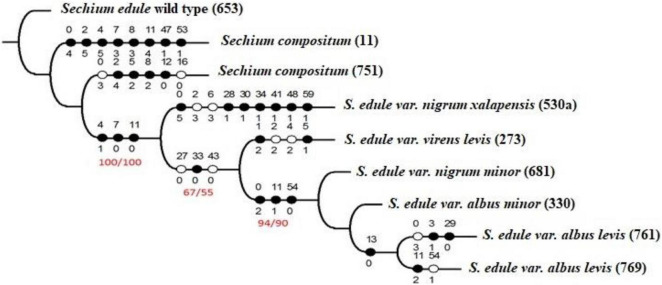
Cladogram with nine genotypes (accessions) of *Sechium* spp., with phytochemical profile values (cucurbitacins and flavonoids), as well as AFLP polymorphic bands in combination with *Eco*RI *ACC and Mse1 CAT*. The white circle corresponds to the new traits, the black circle to the ancestral traits, and the values in the higher part of the circle reveal the trait and lower value of it, in its state. *L* = 87, *Ci* = 72, *Ri* = 38, and values 100/100, 67/55, and 94/90 for the bootstrap/jack knife tests, respectively.

With regard to polymorphic bands associated with phytochemical traits (Figure 2 and Table 5), it is assumed that they are non-coding DNA, neutral to selection. This is why, it is necessary to understand what type of information these bands contain and why they need to be isolated, cloned, and sequenced, and then make an alignment (“blast”) to identify possible associations with biological activity. Although these SMs do not completely determine the flavor of the fruits, they are of great influence. Tables 6, 7 show the concentrations of cucurbitacins and flavonoids per group of genotypes. The sweet fruits showed a sum of 12.59 mg g^–1^, neutral showed 12.00 mg g^–1^, and bitter showed 24.78 mg g^–1^.

**TABLE 6:** Mean concentration of cucurbitacins related to the flavor of the fruit from *Sechium* spp. Values obtained from *n* = 27 ± standard error.

Group	CuD	CuI	CbB	CuE	Σ SMs
Sweet	6.22 ± 3.4	3.24 ± 0.69	1.08 ± 0.32	0.58 ± 0.58	11.12
Neutral	5.15 ± 4.14	5.16 ± 0.57	0.54 ± 0.14	0.00	10.85
Bitter	4.13 ± 2.18	8.43 ± 4.55	2.17 ± 1.21	0.00	14.73

*CuD = cucurbitacin D; CuI = cucurbitacin I; CuB = cucurbitacin B; CuE = cucurbitacin E.*

**TABLE 7:** Mean concentration of flavonoids related to the flavor of fruit from *Sechium* spp. Values obtained from *n* = 27 ± standard error.

Group	Ru	Fz	My	Qu	Na	Ft	Ga	Σ SMs
Sweet	0.21 ± 0.04	0.00	0.67 ± 0.07	0.00	0.05 ± 0.05	0.08 ± 0.02	0.46 ± 0.02	1.47
Neutral	0.15 ± 0.05	0.03 ± 0.03	0.71 ± 0.24	0.0037	0.00	0.11 ± 0.03	0.15 ± 0.15	1.15
Bitter	0.83 ± 0.21	0.36 ± 0.04	0.92 ± 0.37	0.11 ± 0.05	0.72 ± 0.46	1.23 ± 0.62	6.21 ± 3.42	10.05

*Ru = rutin; Fz = florizidin; Mi = myricetin; Qu = quercetin; Na = naringenin; Ft = floretin; Ga = galangin.*

## Discussion

The type of metabolite present in chayote gives each fruit its characteristic flavor. For example, in a previous study (Tallamy and Krischik, 1989), it was mentioned that the qualitative composition of cucurbitacins among species of *Cucurbita* spp. was mutually exclusive. That is to say, the species produces cucurbitacins B and D, or cucurbitacins E and I, with additional qualitative variation in the production of bitter glycosides, where B and D are synthesized mainly as aglicones, while E and I are regularly produced in considerable amounts of glucosides. With *Sechium*, there was no synthesis excluding cucurbitacins per genotype, because D, I, and B appeared in all genotypes, and only E was identified in a sweet accession. This suggests that there is no exclusion of synthesis, but there are differences in terms of concentration.

A previous study (Ríos et al., 2012) mentioned cucurbitacin I (also called JSI-124) as a selective inhibitor of phosphorylate tyrosine JAK3/STAT3, and may be considered a potential anticancer agent in addition to cucurbitacins E and B. It has been previously reported (Takahashi et al., 2009) that cucurbitacin D acts as an inducer of apoptosis in hepatic carcinoma. The evaluated extracts revealed the presence of compounds with possible therapeutic interest, such as cucurbitacins and flavonoids, as reported in different fruits of chayote of separate genetic lineage (Cadena-Iñiguez et al., 2013; Aguiñiga-Sánchez et al., 2017; Salazar-Aguilar et al., 2017), whose antineoplastic and antiproliferative activity has also been previously determined (Petronelli et al., 2009; Bishayeen et al., 2011; Soto-Hernández et al., 2015).

Plants are the excellent source of SM and/or antioxidants. There are compounds that are not of direct use for the survival of plants but help organisms to function more optimally in their environment (Piasecka et al., 2015), such as SMs (O’Connor, 2015) which include phenolics and flavonoids, hydroxybenzoic acids (Sarker and Oba, 2019a), hydroxycinnamic acids (Sarker et al., 2020c), flavanols (Sarker et al., 2020b; Sarker and Oba, 2020b), flavones (Sarker and Oba, 2020e), flavanones (Sarker and Oba, 2020f), tocopherols (Sarker and Oba, 2020d), betalains (Sarker and Oba, 2021), ascorbic acid (Sarker and Oba, 2020c), carotenoids (Sarker and Oba, 2020a), betacyanin (Sarker et al., 2020a), betaxanthin (Sarker and Oba, 2019b), chlorophyll a and b (Sarker et al., 2018a; Sarker and Oba, 2019c), and beta-carotene (Sarker et al., 2018b).

Their production is frequently associated with conditions of biotic and abiotic stress, with the most harmful being the high incidence of light and nutritional deficiencies (Schlaepfer and Mendoza-Espinoza, 2010), thus leading to their carbon-based biosynthesis (phenolic). According to the carbon nutrient balance (CNB) hypothesis, in the case of nitrogen limitation, the SMs lean toward carbon-rich metabolites (phenols and terpenes), and when there is carbon limitation, there is an increase in nitrogen-rich metabolites (alkaloids) (Hamilton et al., 2001; Palumbo et al., 2007).

The literature has shown that any stresses like drought and salinity create reactive oxygen species (ROS) (Sarker and Oba, 2018b), osmotic stress (Sarker and Oba, 2020g), decrease in photosynthetic activities (Sarker and Oba, 2018d), nutrient imbalance (Sarker et al., 2018), and ultimately cause oxidative damage in plant cells (Sarker and Oba, 2018c), that affects the growth and productivity of crops (Sarker and Oba, 2019d). However, to detoxify ROS and cope with stresses, plant evolved mechanisms to augment the concentration of these SMs and/or antioxidants (Sarker and Oba, 2018a).

In this regard, it should be noted that the domesticated and semi-domesticated genotypes of *Sechium* provide nutrients (preferably nitrogenous) in their life history, suggesting an influence on the lower concentration of flavonoids and triterpenes, and, contrary to bitter types, these genotypes may exhibit deficiencies in their wild states, thereby increasing the amount of triterpenes and flavonoids. Hence, stress augments SMs content that can be used in food, cosmetic and pharmaceutical industry.

The probable movement that gave rise to the chayote varieties of *Sechium* spp. highlights the environmental variables and the manipulation that work together to select edible forms. This would promote scenarios for adaptation, where the plasticity of the genus would have mainly allowed morphological and biochemical variations. The semi-domesticated genotypes of yellow fruit express metabolites that can provide carotenoids with greater protection from solar radiation. These fruits are relatively small, which may decrease the area of incidence of radiation and lower the amount of water compared to the domesticated materials, thereby modifying the expression of metabolites that provide protection against predators, such as cucurbitacins (Piasecka et al., 2015).

This is relevant because flavonols show higher protection activity at the level of the cell membrane (López-Revuelta et al., 2006), acting as antiradicals, antichelants, lipid antioxidants, antimutagens, and having an antiproliferative effect, thereby inhibiting carcinogenesis (Martínez-Flórez et al., 2002).

Considering the change in diversity and type of SMs found in the genotypes of *Sechium* spp., it is possible that certain current changes, such as flavor, color, and size, are a direct product of adjustments in the biosynthesis of the mevalonic acid pathway. For example, when photoprotective pigments are required in the fruit, the amount of triterpenes decreases, and with it, the bitter flavor of the fruits, in addition to gradually showing changes in color from dark green to light green with yellow colors of the epidermis. In addition to this, a previous report (Cadena-Iñiguez et al., 2011) described how the ascorbic acid in yellow fruits was attributed to the need for photoprotection, which coincides with the fact that the fruits of edible genotypes of *Sechium* spp. present stomas found in the epidermis (Cadena-Iñiguez et al., 2006). These results assist in explaining the causality of certain morphological variables in *Sechium* spp., in addition to evidencing relevant phytochemical variables that may guide the selection of sweet, neutral, and bitter genotypes, to design gross-breeding.

The cucurbitacins and flavonoids found have demonstrated anti-proliferative, antioxidant, and chelating activity, DNA fragmentation, and apoptosis induction (previously mentioned). However, the evaluated genotypes show contrasts in terms of content and diversity. Some flavonoid and terpenoid derivatives, such as cucurbitacins in one genotype, are at risk of generating intrinsic toxicity (Marinoff et al., 2009), thereby causing long-term intoxication. Therefore, it is important to understand the main phytochemicals and obtain new varieties of *Sechium* showing therapeutic and nutraceutical activity values that are easier to manage for human health and nutrition.

An important fact about the search and identification of natural compounds from species and genotypes little used and that are part of agrobiodiversity, is that when they are not used the trend is their loss in the medium term (Food and Agriculture Organization [FAO], 2019), therefore, this work highlights the opportunity to have alternative sources of inputs for health, pharmacology, and food industry.

## Conclusion

The cladistic analysis can identify and contrast the diversity of cucurbitacins and flavonoids, suggesting that they are related to the polymorphic genetic bands in nine genotypes of *Sechium*, where the bitter flavors (wild) showed a greater number of ancestral traits, which makes them genetically and phytochemically more complex. Flavonoids were present in higher concentrations in genotypes as compared to those of cucurbitacins. However, both groups of metabolites are associated with therapeutic and nutraceutical activity, highlighting genotypes as an important source of biomass and metabolites of interest.

## Data Availability Statement

The raw data supporting the conclusions of this article will be made available by the authors, without undue reservation.

## Author Contributions

JC-I and RS-H: conceptualization. MI-L, RS-H, and KW: investigation. FM-F and MC-C: software, formal analysis, and data curation. MI-L, JC-I, and RS-H: writing – original draft.

## Conflict of Interest

The authors declare that the research was conducted in the absence of any commercial or financial relationships that could be construed as a potential conflict of interest.

## Publisher’s Note

All claims expressed in this article are solely those of the authors and do not necessarily represent those of their affiliated organizations, or those of the publisher, the editors and the reviewers. Any product that may be evaluated in this article, or claim that may be made by its manufacturer, is not guaranteed or endorsed by the publisher.

## Abbreviations

AFLP: amplified fragment length polymorphism
BANGE*Se*: National Germplasm Bank of *Sechium edule*
CuD: cucurbitacin D
CuI: cucurbitacin I
CuB: cucurbitacin B
CuE: cucurbitacin E
Ru: rutin
Fz: florizidin
Mi: myricetin
Qu: quercetin
Na: naringenin
Ft: floretin
Ap: apigenin
Ga: galangin
P388: leukemia in mice
L929: mouse fibroblast cell line
J774: mouse macrophage cell line
HeLa: Henrietta Lacks, human cervical cancer cell line
WEHI-3: leukemic cells.
