# Supplementary figures and images for: Natural Bioactive Compounds of Sechium spp. for Therapeutic and Nutraceutical Supplements
Source: Front Plant Sci. 2021 Dec 21;12:772389. doi: 10.3389/fpls.2021.772389 (PMC9231563; doi:10.3389/fpls.2021.772389)

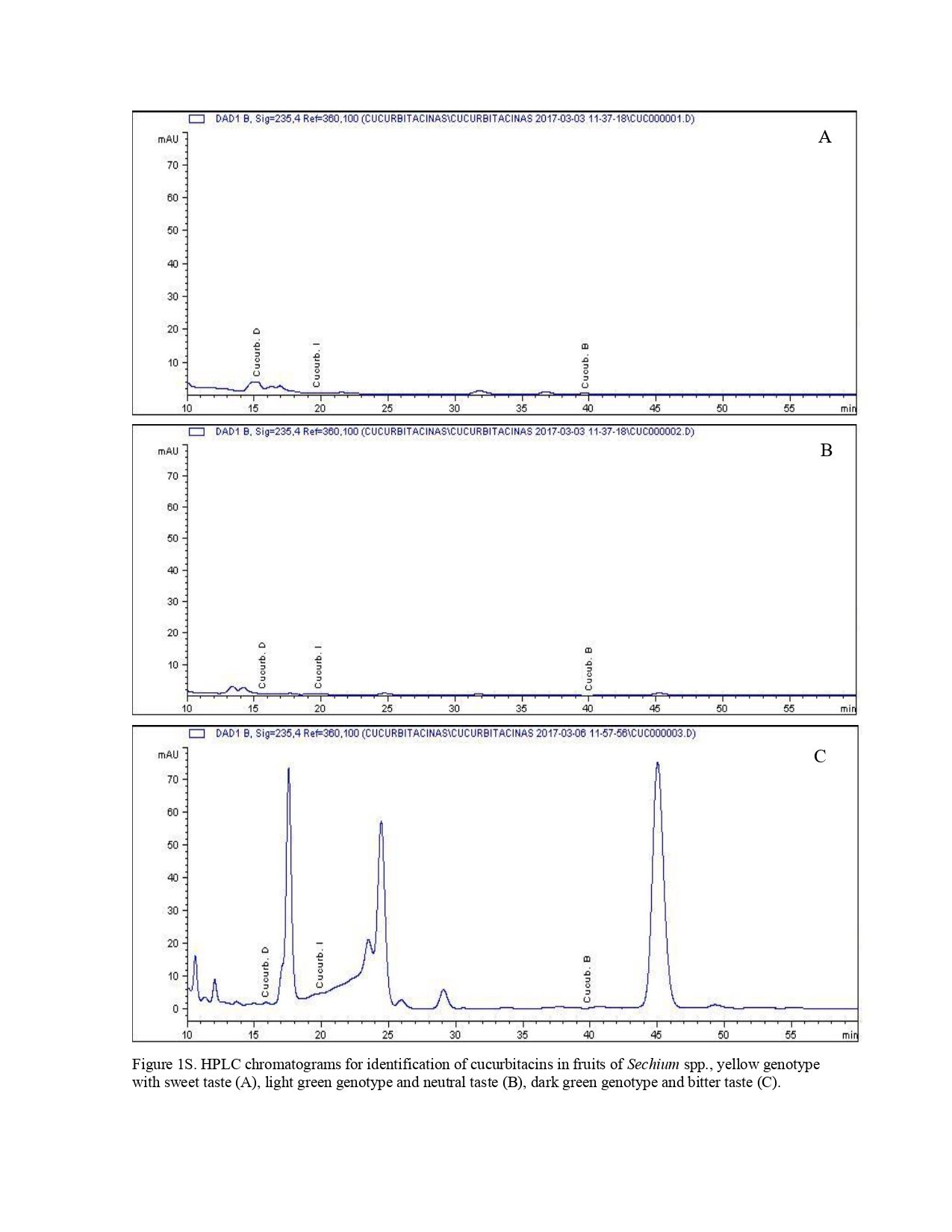

Supplement: Supplementary file 1 [file Image_1.jpg]

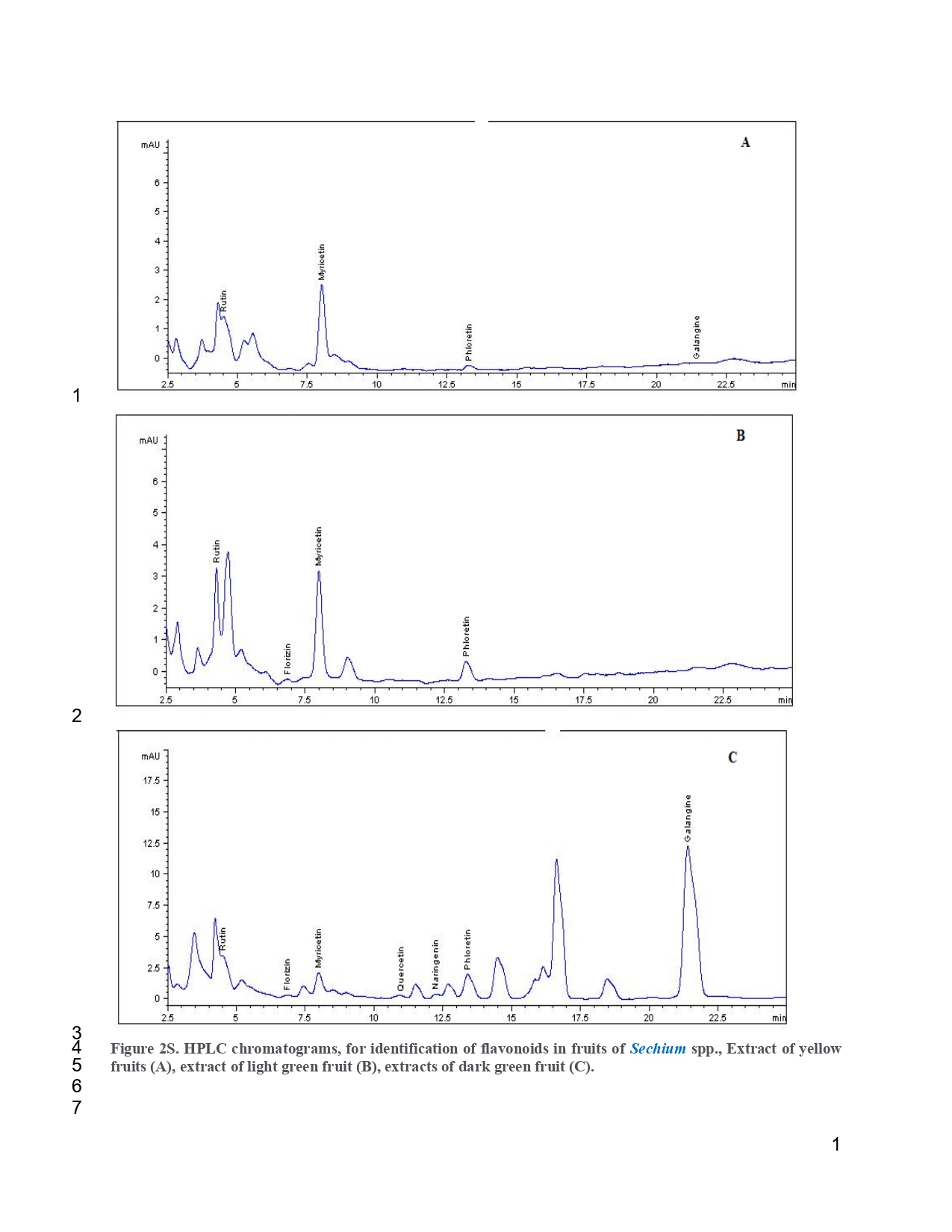

Supplement: Supplementary file 2 [file Image_2.jpg]
